# Summertime Aerosol Composition in Southern Greenlandic Fjords: Impacts of Sources and New Particle Formation Events

**DOI:** 10.1021/acs.est.6c09104

**Published:** 2026-09-28

**Authors:** Mihnea Surdu, Roman Pohorsky, Satoshi Takahama, Julia Schmale

**Affiliations:** † Extreme Environments Research Laboratory, 27218 École Polytechnique Fédérale de Lausanne, Sion 1951, Switzerland; ‡ Laboratory of Environmental Spectrochemistry, 27218 École Polytechnique Fédérale de Lausanne, Lausanne 1015, Switzerland

**Keywords:** Arctic aerosol, chemical composition, new particle formation, source apportionment, NEMS-FTIR, functional groups

## Abstract

Arctic fjord systems are a dynamic interface between ocean, ice, and land, with high summertime biological activity. We present the first aerosol chemical composition measurements from two summer campaigns on the research vessels Forel and Sanna in the fjords near Narsaq, South Greenland, using nanoelectromechanical systems Fourier transform infrared spectroscopy (NEMS-FTIR). Organic matter concentrations (median 1.1 μg m^–3^) exceeded those at High Arctic and midlatitude coastal sites, reflecting the ability of fjords to act as strong aerosol sources. Cluster analysis of FTIR spectra resolved three recurrent aerosol types, with interannual differences linked to phytoplankton bloom timing. To further resolve simultaneous source contributions, positive matrix factorization was applied, identifying a primary marine (53%) and secondary marine (47%) factor, the latter recognized as methanesulfonic acid-containing via characteristic fingerprint-region absorptions. Amine concentrations exceeded those of open-ocean primary marine aerosol, attributed to secondary condensation of gas-phase amines from local sources onto acidic sea spray. High-time-resolution sampling captured chemical composition changes during new particle formation events, with particle number size distributions confirming that such events systematically shift aerosol composition toward a secondary character. These results establish that aerosol in southern Greenlandic fjords is predominantly marine-biogenic and will respond to ongoing glacier retreat and associated changes in fjord productivity.

## Graphical abstract

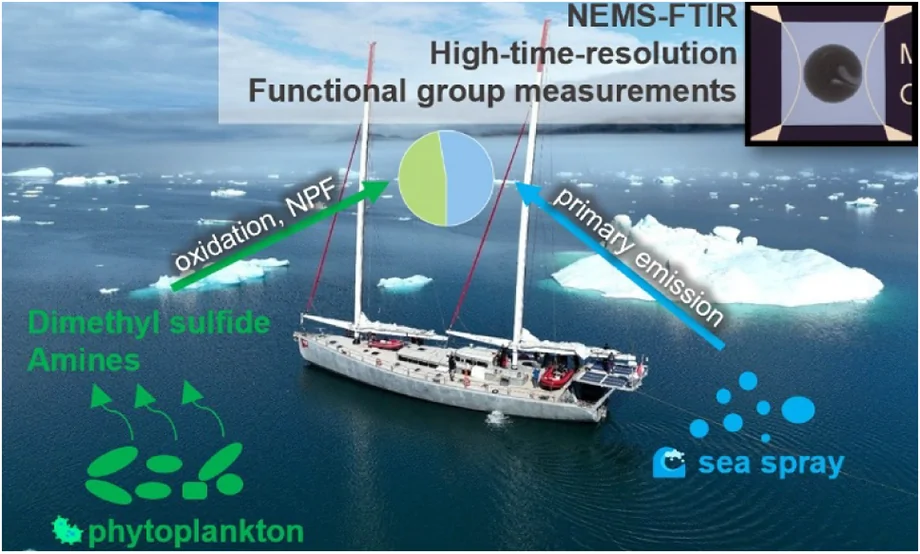

## 1 Introduction

In recent decades, the Arctic has warmed nearly four times faster than the global average.^1^ The Arctic surface energy budget is highly sensitive to cloud radiative effects that are impacted by aerosol,^2^ yet aerosol-cloud interactions in this region remain poorly constrained.^3^ In-situ measurements and characterization of aerosol sources in the Arctic can constrain this uncertainty, as aerosol abundance and chemical composition determine cloud condensation nuclei (CCN) availability which influences cloud formation, phase, lifetime and albedo.^4^

One environment which may be a particularly productive source of climate-relevant aerosol is the fjord, where the ocean, ice and land interface. The extensive Arctic coastline hosts regions of intense biological and chemical activity, particularly during summer.^5^ For example, freshwater melt from marine-terminating glaciers has been found to sustain high marine biological productivity.^6^ This can affect both primary aerosol sources such as sea spray aerosol (SSA)^7^ as well as secondary aerosol by producing emissions of dimethyl sulfide (DMS),^8^ an important precursor for aerosol formation. In addition, coastal seabird colonies provide a source of ammonia which promotes new particle formation.^9,10^ At the same time, coastal settlements^11^ and shipping lanes^5^ may be an important source of anthropogenic aerosol.

The Arctic is highly heterogeneous, making it overly simplistic to generalize aerosol properties across the region. In Greenland, studies of aerosol chemical composition have focused on the high-Arctic Station Nord,^12,13^ Thule (Northwest Greenland)^14^ or Summit station at the top of the Greenland Ice Sheet.^15^ In contrast, southwestern Greenland has a milder climate, with largely sea-ice-free summers that can enhance local biogenic emissions, alongside anthropogenic contributions from the country’s main population centers. It remains unclear whether aerosol concentrations and sources in this region are more comparable to lower-latitude coastal sites or high-Arctic observatories. This not requires not only aerosol abundance data, but detailed chemical composition measurements, which remain scarce in the region.

Across the Arctic, organic aerosols (OA) and sulfate have been identified as the major aerosol mass components,^16^ contributing upward of 75% of the total submicron mass across all seasons.^17^ Nevertheless, OA speciation reveals temporally very different sources, with long-range transported anthropogenic material dominating in winter and biogenic local sources playing an important role in summer.^18,19^ At the same time, global models perform more poorly in the Arctic than at lower latitudes,^20,21^ with only a minority of models considering secondary organic aerosol (SOA).^20^ Indeed, a study using a chemical transport model including aerosol microphysics suggests additional marine SOA sources are needed to reconcile size distribution measurements.^22^ Chemical composition measurements are relatively scarce in the Arctic, therefore additional observations of aerosol chemical composition will help constrain sources and refine models.

High time resolution measurements are needed to relate variations in aerosol chemical composition to process understanding. Currently, such measurements are typically carried out using aerosol mass spectrometry (AMS).^12,13,18^ However, AMS deployment is expensive and logistically challenging, particularly in remote environments such as the Arctic. Alternatively, aerosols can be collected on filters for days-to-weeks, with subsequent offline characterization by either mass spectrometry^19^ or Fourier-transfer infrared spectroscopy (FTIR).^23^ Compared to mass spectrometry, FTIR analysis is cost-efficient and provides information on organic functional groups, which can be used for source signature identification,^24^ hygroscopicity^25^ and volatility^26^ estimations. Recently, we developed specialized nanoelectromechanical systems (NEMS) as substrates for aerosol collection and FTIR analysis, allowing for much lower detection limits and therefore shorter sampling times (minutes-to-hours).^27^ In this study, we use NEMS-FTIR to elucidate the functional group composition and aerosol sources in southern Greenlandic fjords, over two summertime campaigns conducted from research vessels.

## 2 Methods

### 2.1 Campaign Details and Instrumentation

Shipborne measurements were carried out in the fjords around Narsaq, South Greenland, in the summer of 2024 and 2025. The location of the study area and ship tracks can be seen in Figure S1. The 2024 campaign took place from July third to July eighth aboard the Swiss research sailboat Forel and from July 11th to July 21st aboard the Greenlandic research vessel Sanna. In 2025, measurements were made aboard Forel from August 14th to 31st.

During both seasons of the campaign, aerosol was sampled through a Total Suspended Particulates inlet (Digitel AG, Switzerland). Particles between 8 and 286 nm in diameter were measured by a miniaturized Scanning Electrical Mobility Sizer (mSEMS, Brechtel Manufacturing, Inc., USA), consisting of a soft X-ray charger (Model 9002, Brechtel Manufacturing, Inc., USA), a differential mobility analyzer and an advanced Mixing Condensation Particle Counter (amCPC, Brechtel Manufacturing, Inc., USA). Meteorological variables (e.g., solar radiation, temperature, precipitation, wind speed) were measured by an Atmos 41 Gen 2 Weather Station (METER Group, Inc., USA). Aerosols were collected onto NEMS resonators for FTIR analysis using a multichannel filter sampler (FILT, Brechtel Manufacturing, Inc., USA) equipped with filter holders designed to house the NEMS resonators (Aerosol Flow Adapter, Invisible-Light Labs GmbH, Austria). The sampling flow rate was between 0.6 and 0.8 L min^–1^. Aerosols were collected during dedicated sampling periods during which the ship was deliberately oriented such that the air inlet was located upwind of the ship exhaust, in order to avoid exhaust contamination. After sampling, NEMS resonators were stored in sticky-gel carrier boxes and kept frozen until analysis.

NEMS resonators were analyzed using a nanomechanical infrared analyzer (EMILIE, Invisible-Light Labs GmbH, Austria) coupled to an FTIR spectrometer (Invenio R, Bruker Optics, Germany). NEMS-FTIR spectra were recorded in the step scan mode, with the following settings: stabilization delay of 30 ms, 200 coadditions, spectral resolution of 4 cm^–1^, aperture of 6 mm. Spectra were preprocessed and ammonium sulfate and organic functional groups quantified using the approach detailed in Surdu et al.^27^ Briefly, the NEMS-FTIR signal was first converted to absorbance units. Thereafter, the spectral signature of equivalent black carbon was removed by subtracting a spectrum of diesel soot.^28^ Although samples were typically collected during dedicated sampling periods when the air inlet was upwind of the ship exhaust, occasionally the ship’s own exhaust plume was intercepted, for example due to sudden shifts in wind direction or repositioning of the ship. In these cases, black carbon influence was far higher than expected from ambient anthropogenic background levels to the extent that the entire spectrum was distorted (Figure S2). Samples with a high influence of equivalent black carbon were removed from further analysis, considering that they may be affected by ship exhaust. This excluded 14 spectra, leaving 58 spectra in the final data set which was used for all analyses. As shown in Figure S2, polluted spectra are characterized by broad elevated absorbance increasing with higher wavenumber (characteristic of black carbon absorption in the near-infrared), a feature absent from the retained clean spectra, confirming that exhaust contamination does not significantly affect the data set retained for further analysis. Spectra were then baseline corrected using a smoothing splines approach.

Ammonium sulfate and organic functional groups were quantified by fitting specific absorption profiles (lineshapes) to the baselined spectra. The line shape for ammonium sulfate was taken from its complex refractive index reported by Earle et al.^29^ Organic functional groups were fitted using the well-established AIRSpec platform.^30^ This methodology resolves overlapping absorbances by sequentially fitting lineshapes to regions where there are no interferent species absorbing. For example, the interference between carboxylic acid and ammonium absorbance is resolved by first fitting the carboxylic acid line shape to the region around 2600 cm^–1^, where it does not overlap with ammonium. The ammonium sulfate line shape is subsequently fitted to the residual spectrum (i.e., after subtraction of carboxylic acid) in the region between 3000–3100 cm^–1^.

NEMS-FTIR quantification is based on integrated molar absorption coefficients for functional-group classes rather than on calibration against a single molecular species. Molar absorption coefficients from Surdu et al.^27^ were used for ammonium sulfate, alkane, carboxylic acid, alcohol and carbonyl functional groups. The absorption coefficients were obtained by calibration using authentic standard molecules containing each of the functional groups against SMPS-derived particle mass. Uncertainties associated with variability in absorption coefficients and peak fitting were determined to be 20, 9.5, 13 and 31% for alcohol, carboxylic acid, carbonyl and alkane, respectively.^27^ For the amine functional group, the absorption coefficient was obtained from the calibration of Liu et al.^31^ which used three primary amine standards (uncertainty was not reported by the authors). The moles of analyte on the resonator were converted to ambient mass concentrations using the volume of air filtered and a size-dependent collection efficiency calculated using the collocated mSEMS particle number size distributions.^27^ Organic matter was calculated as the sum of the quantified functional group mass concentrations. Oxygen-to-carbon ratios (O:C) of organic matter were estimated by relating each functional group to its associated carbon and oxygen atoms, following the approach of Takahama and Ruggeri (2017),^32^ taking into account nonfunctionalized carbon atoms are not directly detectable by FTIR.

### 2.2 Clustering of NEMS-FTIR Spectra

Hierarchical cluster analysis was used to group NEMS-FTIR spectra by spectral similarity. Wavenumbers between 4000 and 875 cm^–1^ were included. Spectra were smoothed using a Savitzky-Golay filter (first order). Spectra were then L2-normalized such that the square root of the sum of squared intensities equals 1, preserving spectral shape (chemical composition) while removing overall intensity (mass loading) differences between spectra. Dimensionality reduction was then performed using principal component analysis on the spectral vectors (absorbance value for each wavenumber), retaining the number of components required to explain >99% of the variance. The correlation metric using average linkage was used for clustering spectra using the principal component scores obtained in the previous step. The number of clusters was selected to be three based on the minimum number of samples per cluster, Silhouette coefficient and within-cluster sum of squares (Figure S3). Although selecting four clusters would reduce the within-cluster sum of squares and slightly increase the Silhouette coefficient, the resulting fourth cluster was a small cluster (6 spectra) differentiated mostly by alkane peaks. Therefore, three clusters were chosen as the ideal solution.

### 2.3 Positive Matrix Factorization (PMF) of NEMS-FTIR Spectra

As each measured spectrum reflects the simultaneous contributions of multiple aerosol sources, cluster analysis assigns the spectrum as a whole to a single cluster but does not resolve the underlying source contributions. Therefore, PMF analysis was applied to separate and quantify the individual source factor profiles. PMF inputs were NEMS-FTIR spectra in units of absorbance with wavenumbers in the range 4000–875 cm^–1^ at a resolution of 2 cm^–1^. Analysis was performed using SoFi Pro (Datalystica, Switzerland). The error matrix consisted of the standard deviations, as a function of wavenumber, averaged from 24 empty NEMS resonator measurements. Two to six factor solutions were examined, with rotational (FPEAK) values between −1.5 and 1.5 (step change of 0.5). The explained variance as well as Q/Q_exp_ decreased with increasing factor number (Figure S4). However, solutions with 3 or more factors resulted in unrealistic spectra. Such profiles featured either absorption from only single functional groups, or profiles lacking absorption due to the alkane functional group, similar to previously observed factor splitting.^33,34^ Therefore, the 2-factor solution was selected. An FPEAK value of 0 was used, corresponding to a minimum in Q/Q_exp_, however factors were observed to be insensitive to FPEAK. Averaging over the entire campaign, the selected 2-factor solution explained approximately 75% of the total variance in the measured absorbance. The output solution was verified to be robust to variations in spectral smoothing, downsampling (reducing spectral resolution by using fewer wavenumber points) or downscaling (reducing influence of specific wavenumber regions by increasing their uncertainties).

## 3 Results and Discussion

### 3.1 Campaign Overview

The 2024 and 2025 campaigns captured a broad range of aerosol conditions over the measurement period, as illustrated in Figure 1. Frequent new particle formation events were observed, consistent with our previous measurements in the area^11^ (Figure 1a). Using NEMS-FTIR, 59 aerosol samples were used to quantify organic functional groups and ammonium sulfate (Figure 1b). Overall, the median concentration of organic matter (as the sum of quantified NEMS-FTIR functional groups) and ammonium sulfate was 1.1 μg m^–3^. The main functional groups present were alkane [median ± interquartile range (IQR) of 290 ± 340 ng m^–3^], alcohol (230 ± 210 ng m^–3^) and amine (210 ± 240 ng m^–3^). The ammonium sulfate concentration was 200 ± 240 ng m^–3^. The aerosol was highly oxygenated, with a mean oxygen-to-carbon ratio (O:C) of 0.69.

**Figure 1:**
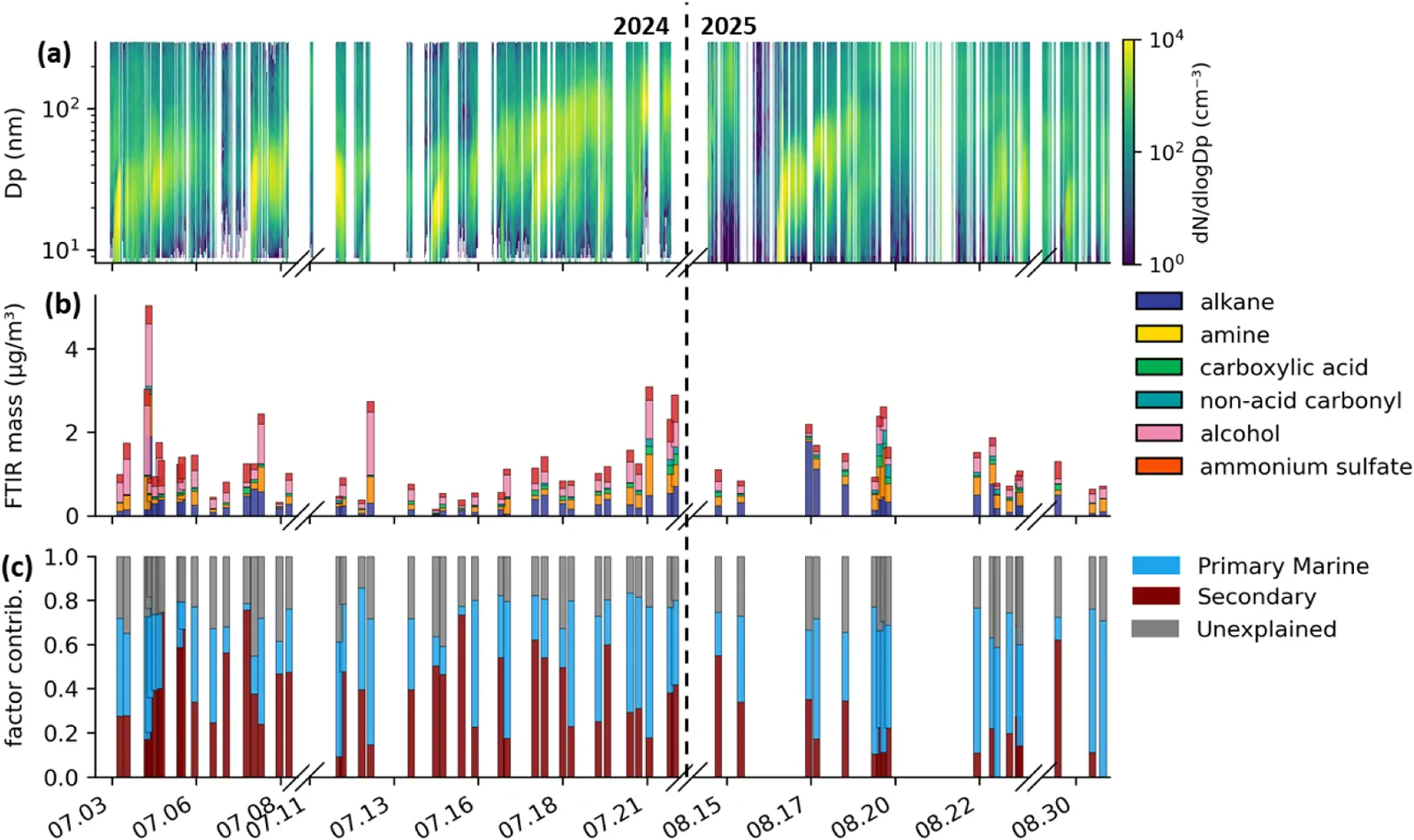
Time series for the 2024 and 2025 campaigns. Particle number size distributions in panel (a). Data gaps correspond to either time periods contaminated by ship exhaust which were filtered out or periods affected by instrumental issues. Quantified functional group composition from NEMS-FTIR in panel (b). Panel (c) shows the contribution of the PMF factors identified, as well as the unexplained variance.

Comparing the two years, the O:C is significantly different (p-value 0.02, Mann–Whitney u-test) between 2024 (median 0.71) and 2025 (median 0.54). Similarly, significant (*p* < 0.05) differences between years are observed for alcohol, amine and nonacid carbonyl functional groups. These differences may be related to varying aerosol sources between the years, which were analyzed using PMF (Figure 1c and section 3.3) and cluster analysis.

### 3.2 Cluster Analysis

Cluster analysis of the FTIR spectra can group samples with similar chemical composition, and therefore presumably sources and atmospheric processing. A three-cluster solution was found to be optimal (see Methods). The clusters separate both in terms of ammonium sulfate and organic functional group abundance (Figure 2). The OM is rich in alcohols and amines for all clusters, which is characteristic of primary marine particles from sea spray.^35^ The alcohol fraction of OM is 0.30 ± 0.18 (median ± IQR), which is somewhat smaller than that of primary marine particles over the open ocean^35^ (0.65 ± 0.12) or artificially produced from bubbled seawater^35^ (0.55 ± 0.14). This observation suggests differing emission sources in the fjords compared to the open ocean, and/or further atmospheric processing.

**Figure 2:**
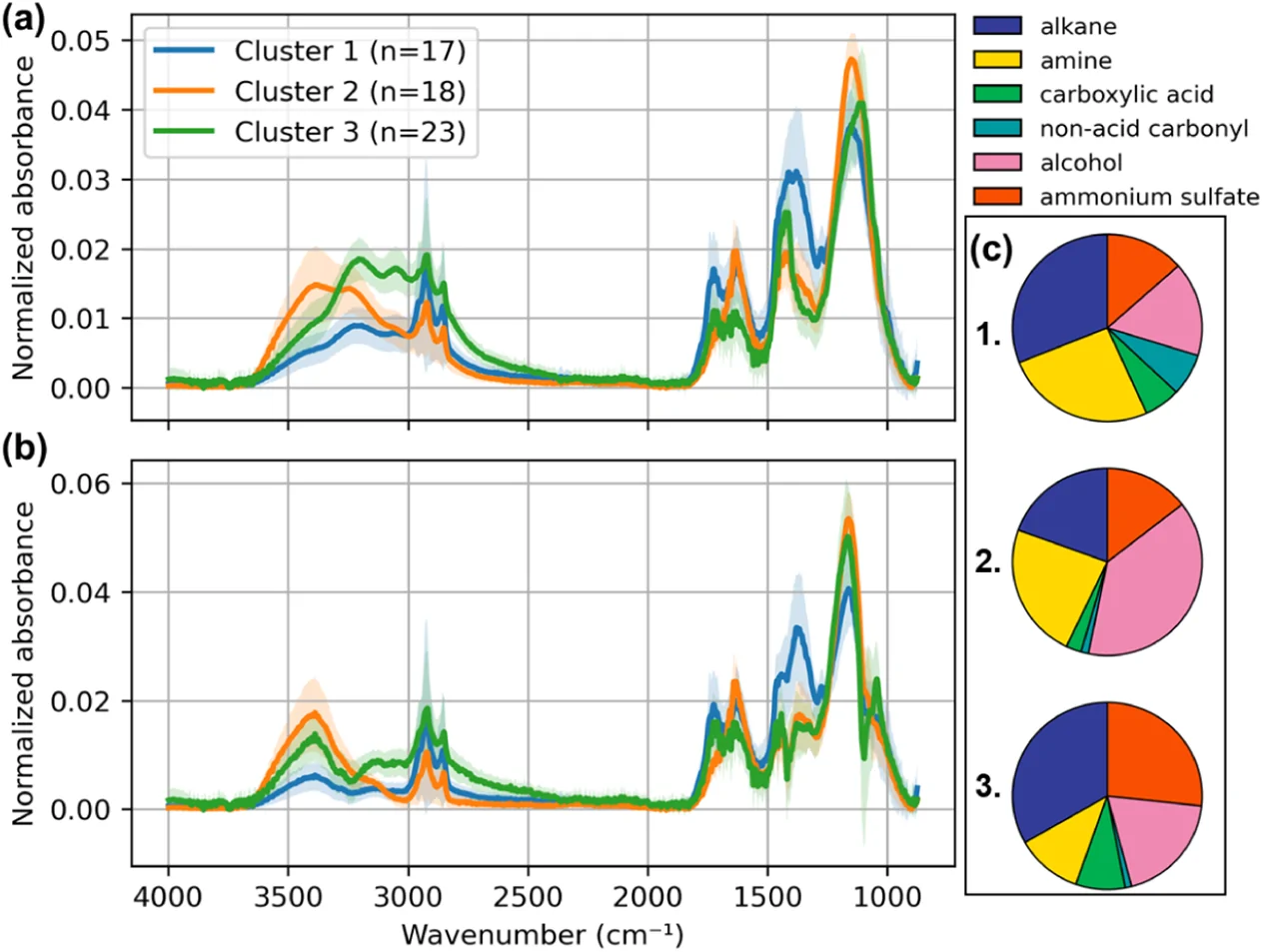
Cluster analysis and relative chemical composition of NEMS-FTIR spectra. Panel (a) shows full spectra, while panel (b) shows spectra with ammonium sulfate absorbance subtracted. Organic functional group and ammonium sulfate contributions to each cluster are shown in panel (c).

The O:C of the three clusters is 0.66, 0.90 and 0.57, respectively. However, in marine conditions, it is not appropriate to use O:C as a metric of atmospheric aging, since the high abundance of alcohols from saccharides in primary marine aerosol also leads to high O:C values.^35^ Alternatively, we use the ratio of alcohols to the sum of carboxylic acids and nonacid carbonyls as a proxy for atmospheric aging, providing a clear separation of the clusters (Figure 3a). Cluster 2 is most similar to open-ocean primary marine particles (alcohol fraction 0.45 ± 0.17, low carboxylic acid and carbonyl abundance), while clusters 1 and 3 show relatively higher carboxylic acid and nonacid carbonyl contributions. Clusters 1 and 3 are therefore more likely to have secondary aerosol contributions or have undergone atmospheric aging. Indeed, Cluster 3 is relatively richer in ammonium sulfate (26% of NEMS-FTIR detectable mass, i.e., organic matter + ammonium sulfate), suggesting contributions from secondary aerosol to this cluster.

**Figure 3:**
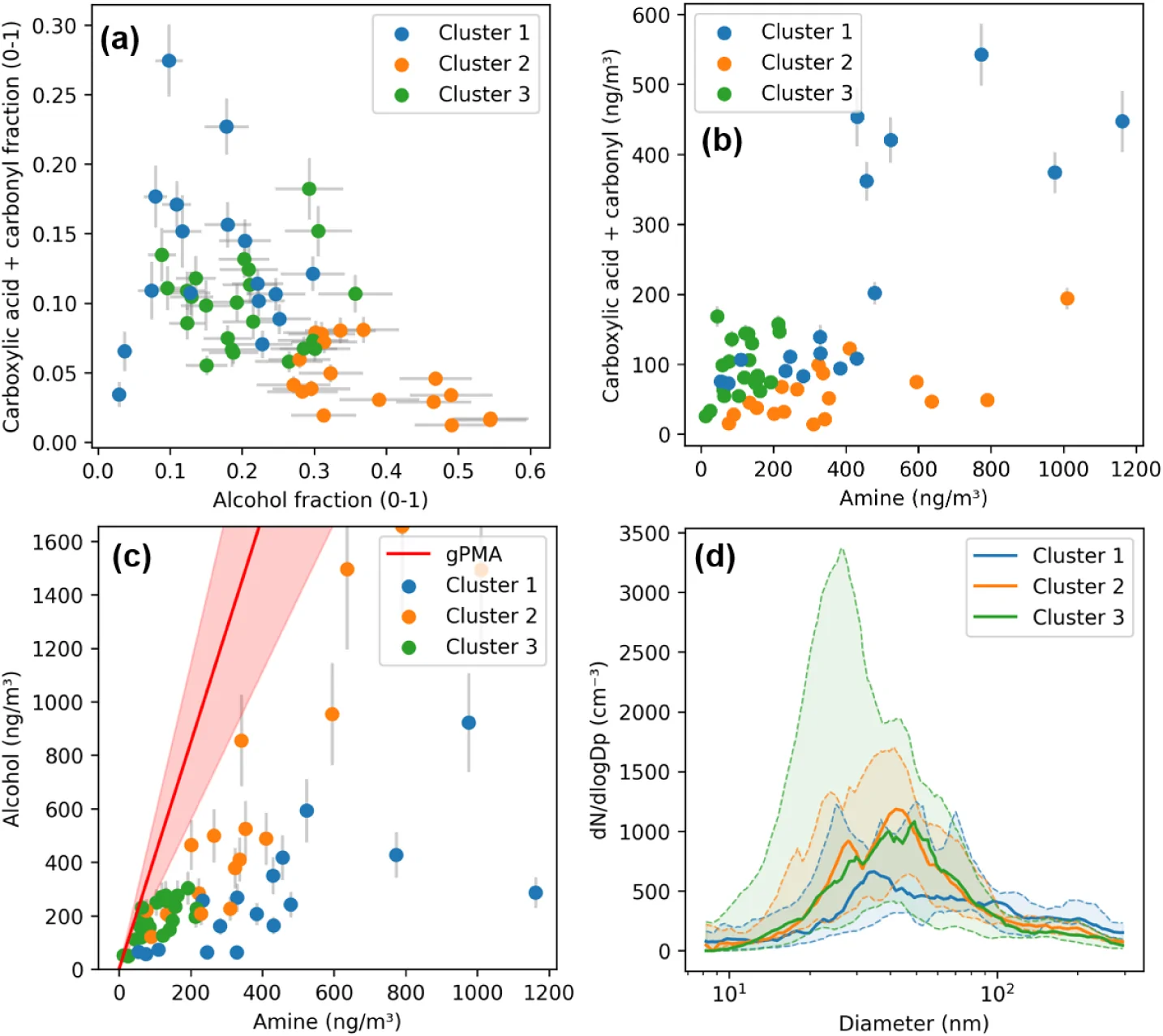
Scatter plot of fractional contribution of carboxylic acid and nonacid carbonyl versus the alcohol functional group in panel (a). Data points are colored by the cluster that they belong to. Panel (b) shows a scatter plot of carboxylic acid and carbonyl versus amine concentrations. Panel (c) shows a scatter plot of alcohol versus amine concentrations. gPMA refers to data from primary marine aerosol artificially generated from bubbling seawater from the study of Frossard et al.^35^ The solid line represents the mean and the shaded area one standard deviation. Error bars in panels (a–c) represent propagated uncertainties from quantification of each of the functional groups, as detailed in the Methods section. Panel (d) features particle number size distributions associated with each cluster. Solid lines represent the median while the shaded area between dashed lines represents the 25–75th percentile range.

Remarkably, all three clusters have a large contribution from amine functional groups (22 ± 16% of OM, median ± IQR). Primary marine particles have been found to have a substantial amine component^35^ (6 ± 6% of open-ocean and 13 ± 3% of seawater-bubbled OM) but generally lower than our observations. Hence, the increased amine fractions in our measurements suggest a secondary contribution. Indeed, we find a good correlation (Pearson correlation coefficient of 0.61) between amines and functional groups typical of SOA like carboxylic acids and nonacid carbonyls, supporting a secondary amine component (Figure 3b). Such correlations have also been observed by a recent study in the North Atlantic, linked to a secondary biogenic ocean source.^36^ Alternatively, overlapping absorbances from carboxylates (conjugate bases of carboxylic acids) may contribute to the FTIR signal attributed to amines. The amine functional group was quantified by integrating the peak centered at 1625 cm^–1^, corresponding to primary (i.e., R–NH_2_) amine N–H bending, as in previous literature.^31,37^ However, carboxylates also absorb in this region, with aliphatic monocarboxylates absorbing between 1541–1676 cm^–1^,^38^ meaning some absorbance attributed to amines may in fact arise from overlapping carboxylate peaks.

The observations of the high “amine” peak at around 1630 cm^–1^ in all clusters may be explained using particle pH and site-specific sources. A recent study used controlled experiments to determine that sea spray aerosol becomes acidic within minutes of formation, reaching values of 2–4 pH units, with smaller (submicron) particles being more acidic.^39^ Similar observations were also made at mid- and high-latitude locations.^40–42^ Under these acidic conditions, organic acids with typical p*K*_a_ values above 4 would mostly be in their protonated form. Therefore, the carbonyl absorption would be present upward of 1680 cm^–1^ rather than contributing to the elevated peak at 1630 cm^–1^. More importantly, basic amines in the gas phase could readily be taken up onto the acidic sea spray aerosols and absorb in the region of 1630 cm^–1^. Similarly to the primary sea spray particles, basic gas-phase amines could also condense on sulfate-rich particles such as those in Cluster 3. The elevated 1630 cm^–1^ amine peak was not observed in the previous study of sea spray aerosol FTIR characterization by Frossard et al.,^35^ which may be related to the fact the authors focused on open-ocean aerosol, with continental emissions deliberately being filtered out. Indeed, amines have a significant source from livestock farming^43^ and the Narsaq region is abundant in farming activity, hosting 68% of Greenland’s arable land used for livestock grazing (mostly sheep).^44^ In addition to the anthropogenic amine source, high biological productivity driven by nutrients from freshwater melt in the fjord^6^ can also increase biogenic oceanic amine emissions.^45^ We therefore hypothesize that elevated gas-phase amine concentrations in the fjord system, which are not present over the open ocean, lead to SOA by condensation onto acidic sea-spray aerosols as well as sulfate-containing particles. Still, we cannot completely rule out carboxylate contribution as some small diacids with low p*K*_a_ values such as oxalic (p*K*_a1_ = 1.25) and malonic acid (p*K*_a1_ = 2.83) may be found in their monocarboxylate form even at pH 2–4, and indeed lead to increased absorption at 1630 cm^–1^. To quantitatively test this possibility, we performed a sensitivity analysis fixing the amine contribution to that of primary sea spray aerosol and assigning excess 1630 cm^–1^ absorbance to carboxylates (Supplementary Text S1, Figure S5). This scenario appears unlikely, as it requires a campaign-average carboxylic acid + carboxylate contribution of 25% of organic matter, significantly higher than reported at other marine and Arctic sites. Moreover, it would require carboxylate concentrations to be on average 2.8 times higher than carboxylic acids.

While all clusters feature a large concentration of amines, the ratio of alcohol to amine FGs can help inform the extent to which the clusters are made up of primary particles, since the alcohol/amine ratio has been found to be relatively constant in primary marine particles.^35^ Cluster 2 features an alcohol/amine ratio similar to that of primary marine aerosol artificially generated from bubbling seawater (gPMA), suggesting a mainly primary origin (Figure 3c). On the other hand, Cluster 1 is depleted in alcohols over amines, suggesting further oxidation of alcohols. This is consistent with the increased fraction of carboxylic acids and carbonyls in Cluster 1.

In terms of temporal distribution, Clusters 2 and 3 dominate the July 2024 campaign, whereas Cluster 1 is the main cluster present in August 2025 (Figure S6). Still, all three clusters are present in both campaigns. No statistically significant differences in meteorological variables (radiation, temperature, precipitation or wind speed, Figure S7) associated with the three clusters are observed, suggesting that interannual differences are instead driven by source variability. Indeed, chlorophyll-a concentrations are higher in July 2024 than in August 2025 (Figure S8), as the 2024 campaign date is closer in time to the seasonal phytoplankton bloom. Biogenic marine emissions, including DMS, are therefore likely elevated in July relative to August, leading to an increased contribution of Cluster 3 containing a higher fraction of ammonium sulfate, produced by DMS oxidation. In August 2025, with comparatively lower DMS emissions but broadly similar photochemical activity between the two months, atmospheric oxidation of primary sea spray particles is a more dominant oxidation pathway, possibly explaining the more aged character of Cluster 1 that is prevalent during that period.

Cluster 3 is associated with elevated Aitken mode particle number concentrations, with a mode around 25 nm (Figure 3d). During both 2024 and 2025 campaigns, frequent new particle formation and growth events (NPF) were observed in the particle number size distributions. NPF events are typically associated with elevated concentrations of (extremely) low-volatility gaseous compounds. Besides nucleating and forming new particles, they can modify the chemical composition of preexisting accumulation mode particles through condensational growth.^46^ Both nucleation and condensational growth would transfer the chemical character of secondary aerosol, namely sulfate and oxidized organics, into the bulk chemical composition detectable by NEMS-FTIR. Although these vapor concentrations were not directly measured in this study, the association of Cluster 3, which carries strong secondary signatures of ammonium sulfate, carboxylic acids, and carbonyls, with elevated Aitken mode concentrations therefore suggests that frequent NPF in the region is capable of altering the bulk chemical composition. Our previous study in the region used particle number size distributions to show that NPF affects background aerosol conditions^11^ by modulating the Aitken mode number concentrations. The current work provides complementary chemical evidence for the role of NPF in shaping background aerosol composition.

Overall, the three clusters contribute roughly equally to the NEMS-FTIR detected mass at 36, 35 and 28%, respectively. There is evidence of primary sea spray emissions in all three clusters, identified by substantial alcohol and amine FGs, characteristic of saccharide-rich sea spray. At the same time, all clusters also carry secondary characteristics, including ammonium sulfate and amines from secondary sources, with additional carboxylic acid and carbonyl groups in Clusters 1 and 3. As sampled aerosol likely reflects contributions from multiple sources simultaneously, cluster analysis alone cannot separate the underlying source contributions. PMF analysis is therefore applied in the following section to separate and quantify the source factor profiles.

### 3.3 PMF Analysis

The two factors from the PMF analysis were identified by their characteristic spectra and similarity to previous literature spectra as well as chemical standards. The first factor is assigned to primary marine aerosols originating from sea spray, due to the strong similarity to atmospheric primary marine aerosol (aPMA) observed by Frossard et al. (2014)^35^ (Figure 4a). There is good agreement between the spectra in regard to the alcohol peaks (around 3400 cm^–1^) and alkane peaks (2920 and 2855 cm^–1^). Compared to the literature aPMA spectrum, the factor observed in this work has a higher primary amine peak at 1630 cm^–1^. This is attributed to gas-phase amines, from both marine and animal husbandry sources in the area, rapidly condensing on acidic sea spray particles as discussed in the previous section.

**Figure 4:**
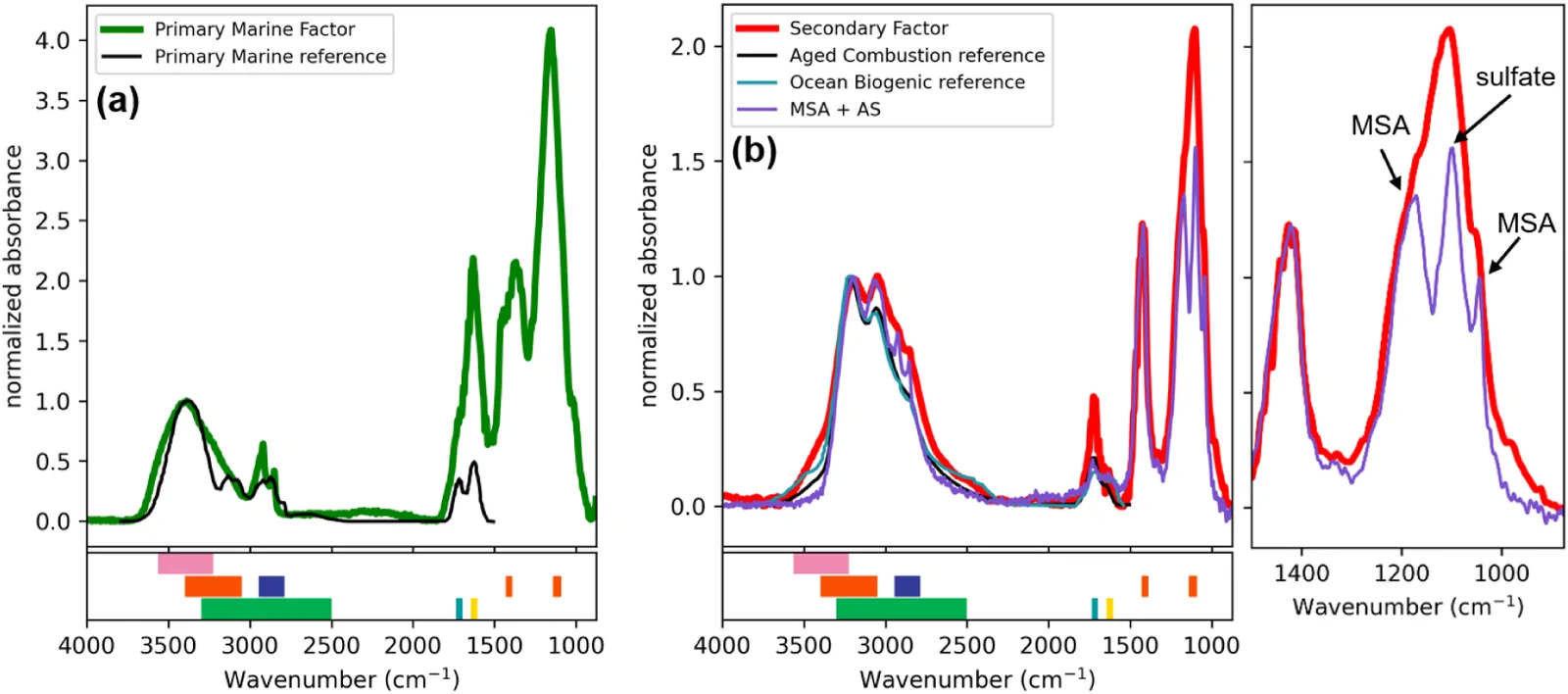
PMF factor results from this study compared to previous literature and reference compounds. Panel (a) compares the primary marine factor to that of Frossard et al. (2014).^35^ Panel (b) compares the secondary factor from this work to the aged combustion factor from Xia et al. (2022)^47^ and ocean biogenic factor (low O:C) from Russell et al. (2011).^24^ A comparison is also made to the linear combination of spectra of standard pure methanesulfonic acid and ammonium sulfate (MSA + AS), with an inset zooming in on the fingerprint region. The horizontal bars below the panels represent the wavenumber ranges used for quantification of the different functional groups, using the color legend in Figure 2.

The second factor identified is a secondary factor, attributed as such due to its high contributions from atmospherically aged functional groups (nonacid carbonyls and carboxylic acids) and ammonium sulfate. These features are visible in the spectra as a tall peak at 1715 cm^–1^ (nonacid carbonyl), a shoulder at 2700 cm^–1^ (carboxylic acid), tall peaks at 1420 and 1100 cm^–1^ (ammonium and sulfate, respectively) as well as complex broad absorption in the region 3250–2900 cm^–1^ (ammonium). The factor is similar to both aged fossil-fuel combustion profiles^47^ as well as a previous ocean biogenic factor^24^ (Figure 4b). This similarity likely reflects the substantial contribution of ammonium sulfate in all three profiles. Sulfate formed from biogenic marine sulfur precursors (e.g., DMS oxidation), and subsequently neutralized by ammonia is chemically indistinguishable from anthropogenically derived ammonium sulfate, making source attribution challenging and meaning we cannot rule out possible contributions from anthropogenic sulfate. Therefore, the similarity between this factor and previously reported marine and aged combustion factors likely arises from the presence of ammonium sulfate in all factors, in addition to atmospheric processing which produces carboxylic acid and nonacid carbonyl functional groups.

Unlike FTIR analysis of aerosols collected on PTFE filters, where PTFE interference may complicate interpretation in the fingerprint region of the spectrum (<1500 cm^–1^), NEMS-FTIR does not suffer from this limitation. Therefore, peaks in the fingerprint region are used here to identify the presence of methanesulfonic acid (MSA), an oxidation product of DMS. A linear combination of the spectra of pure MSA and ammonium sulfate was scaled to the secondary aerosol factor profile (Figure 4b and inset). A very good match was observed between the factor profile and the scaled spectrum. In particular, a set of three distinct peaks (1175, 1100, and 1050 cm^–1^) in the fingerprint region are common to both spectra. The 1100 cm^–1^ peak is assigned to sulfate^29^ whereas the other two are MSA-related, with previous work analyzing MSA salt in solution finding the dominant peaks to be located at 1185 and 1048 cm^–1^.^48^ This cluster of three peaks is not present in the primary marine factor, where the major peak in the region is the peak at 1155 cm^–1^, likely associated with C–O vibration of alcohol groups (Figure S9). The spectral similarity to MSA therefore suggests a strong biogenic contribution (secondary marine) to this factor, however a minor anthropogenic component cannot be excluded due to the chemical identity of anthropogenic and biogenic ammonium sulfate.

The primary and secondary marine factors contribute 53% and 47%, respectively, to the total NEMS-FTIR detected aerosol mass (sum of organic functional groups and ammonium sulfate) in the region. The factor analysis suggests that aerosol sources in the region are of mostly biogenic (marine) origin. Nevertheless, we cannot exclude anthropogenic/terrestrial contributions to the secondary factor, since the substantial ammonium sulfate component is spectroscopically similar regardless of whether the sulfate originates from marine biogenic or anthropogenic sources. Combining the factor analysis and clustering results (Figure S10), we observe that clusters 1 and 2 are predominantly primary marine in origin, with median 73 and 66% contribution of the primary marine factor, respectively. Conversely, cluster 3 is largely (median 74%) attributed to the secondary marine factor, as hypothesized earlier due to the large contribution of ammonium sulfate to the cluster. This is consistent with cluster 3 being associated with NPF events as seen by the elevated Aitken mode in the particle number size distribution present during this cluster.

### 3.4 Case Study: High-Time-Resolution Chemical Composition Measurements

On July fourth 2024, multiple NEMS-FTIR measurements were made during a day which featured an NPF event, highlighting the potential of NEMS-FTIR for high-time-resolution measurements of aerosol chemical composition. During the NPF event, the particle number concentration increases from several hundred particles cm^–3^ up to 4000 cm^–3^. The new particle mode grows up to 30 nm in size, while the preexisting particle mode also increases in size from around 40 to 80 nm (Figure 5a). At the same time, the PMF analysis shows that the contribution of the secondary marine factor increases from 23% at the start to 100% by the end of the NPF event (Figure 5b). As the NPF event progresses, the NEMS-FTIR spectra show a relative decrease in the alcohol and amine functional groups, with peaks at around 3400 and 1630 cm^–1^, respectively (Figure 5c). At the same time, gradual increases in carboxylic acids (2700 cm^–1^), ammonium (3250–2900 and 1420 cm^–1^) and sulfate (1100 cm^–1^) are observed.

**Figure 5:**
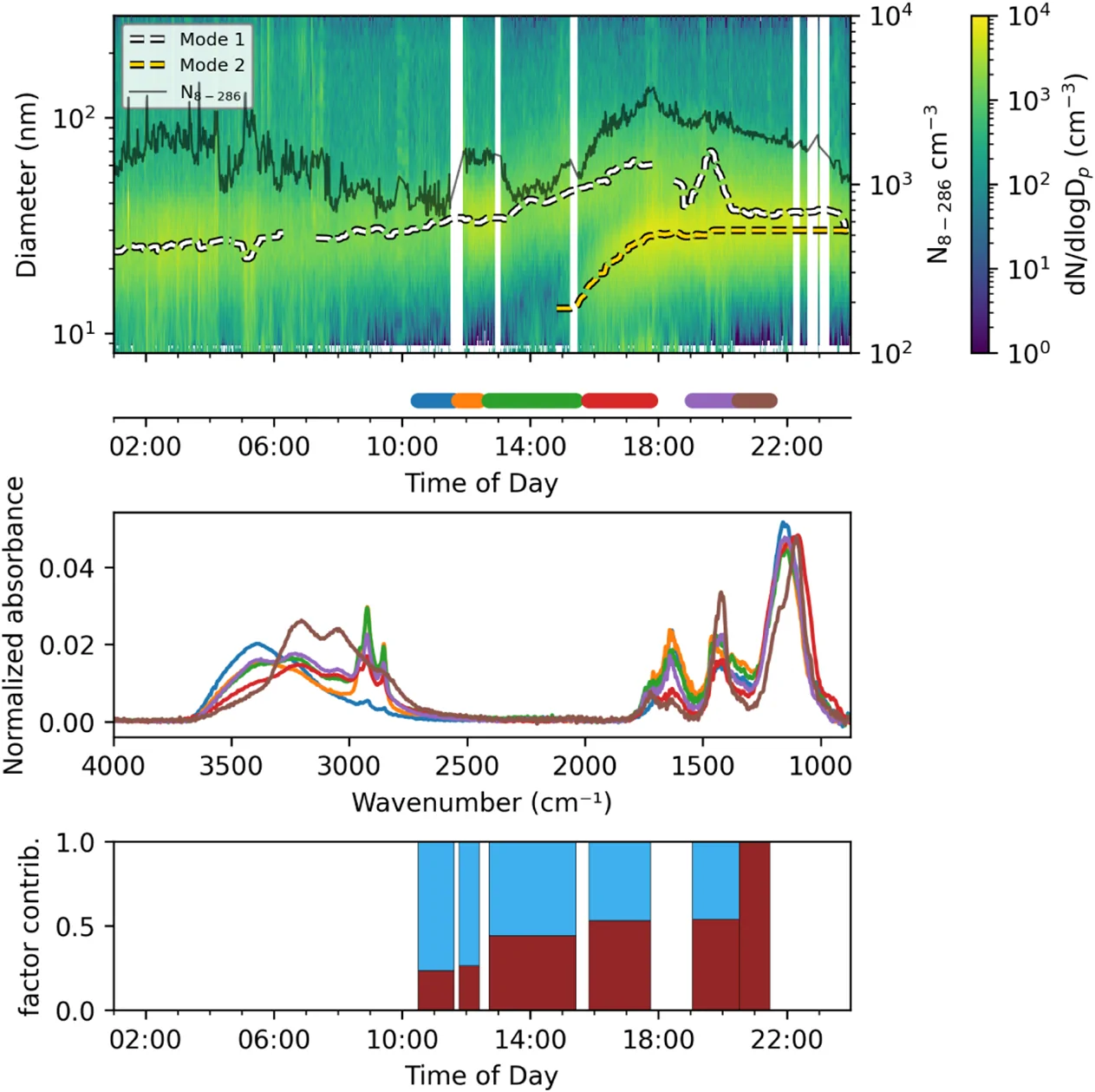
Case study of chemical composition change during an NPF event. The onset of the new particle formation is observable around 13:00 with our instruments as an elevated concentration of particles in the size range (8–20 nm), coinciding with the start of the preexisting particle mode growing in size. Panel (a) shows the particle number size distribution and mode diameters (left axis) as well as total number concentration of 8–286 nm particles (right axis). Inset below panel (a) represents sampling times of aerosols onto NEMS resonators, using the same color coding as the NEMS-FTIR spectra shown in panel (b). Factor contribution of PMF factors is shown in panel (c).

Besides the monotonic increase in the secondary factor fraction during the NPF event, small but detectable variations not captured by the PMF analysis provide additional insights into chemical transformations taking place. These variations are highlighted by difference spectra among consecutive samples (Figure S11). For example, the spectrum of the sample collected at the tail end of the growth event (15:48–17:44) features higher contributions from organics (O–H stretch from alcohols or hydroperoxides around 3400 cm^–1^, and peaks near 1650–1750 cm^–1^ indicative of carboxylic acids and amines) than the preceding spectrum. This may be a direct observation of the established pathway where sulfate is a dominant condensing species for small particles (where extremely low volatility components are needed to overcome the Kelvin effect), and higher volatility organics are later able to contribute to growth by condensation as particle size increases.^49^ Changes in air mass or emission profiles due to changes in meteorological factors may also account for some of the variations, particularly in the sample (11:46–12:24, orange) where a prominent increase in aliphatic C–H accompanying a shift in particle size distribution is observed over the previous sampling interval.

This case study provides a direct observation of how NPF events affect the bulk aerosol chemical composition in the region. Although the contribution of the newly formed particles to total aerosol mass is likely small (due to their small size), condensation of low-volatility gaseous compounds (present at elevated concentrations during NPF) onto the preexisting particle population can explain the observed compositional shift, as has been observed elsewhere.^46^ Here, the gradual change in chemical composition was determinable due to the high sensitivity of NEMS-FTIR, allowing for high-time-resolution sampling (sampling duration 1–2 h). This is unachievable with conventional filter sampling (e.g., PTFE-FTIR, offline AMS), which requires longer sampling times. While this observation represents a single case study (intraday repeated high-time-resolution measurements were rarely conducted during our campaign), it is corroborated by two additional pieces of evidence. The association between Cluster 3 (comprising 74% secondary marine factor) and Aitken mode particles suggests a systematic link. In addition, previous ground-based observations in Narsaq show that NPF and growth events substantially contribute to the background particle number size distribution in the region.^11^

### 3.5 Comparison to Arctic and Marine Sites

The total OA mass concentrations in the Narsaq region are relatively high in the context of polar regions (median 1.11 μg m^–3^), substantially exceeding most high Arctic sites as well as midlatitude sites (Figure 6, bar chart, and Supporting Text S1). This observation likely reflects the unique, yet understudied, character of fjord environments, where high biological productivity and sea-spray generation produce high aerosol mass concentrations. In terms of OA chemical composition, the measurements at Narsaq are very comparable to AMS measurements at Mace Head, a midlatitude site off the coast of Ireland (Figure 6).^50^ In both Narsaq and Mace Head, a large fraction of OA is from sea spray primary production (53 and 42%, respectively). At Mace Head, secondary factors pertaining to MSA-related OA (17%) and more oxygenated OA (OOA, 32%, attributed to atmospherically aged organics from both marine or terrestrial origin with a large fraction of carboxylic acids) are observed, both of which are likely incorporated in the Secondary factor identified in this work (47%), as discussed in section 3.3.

**Figure 6:**
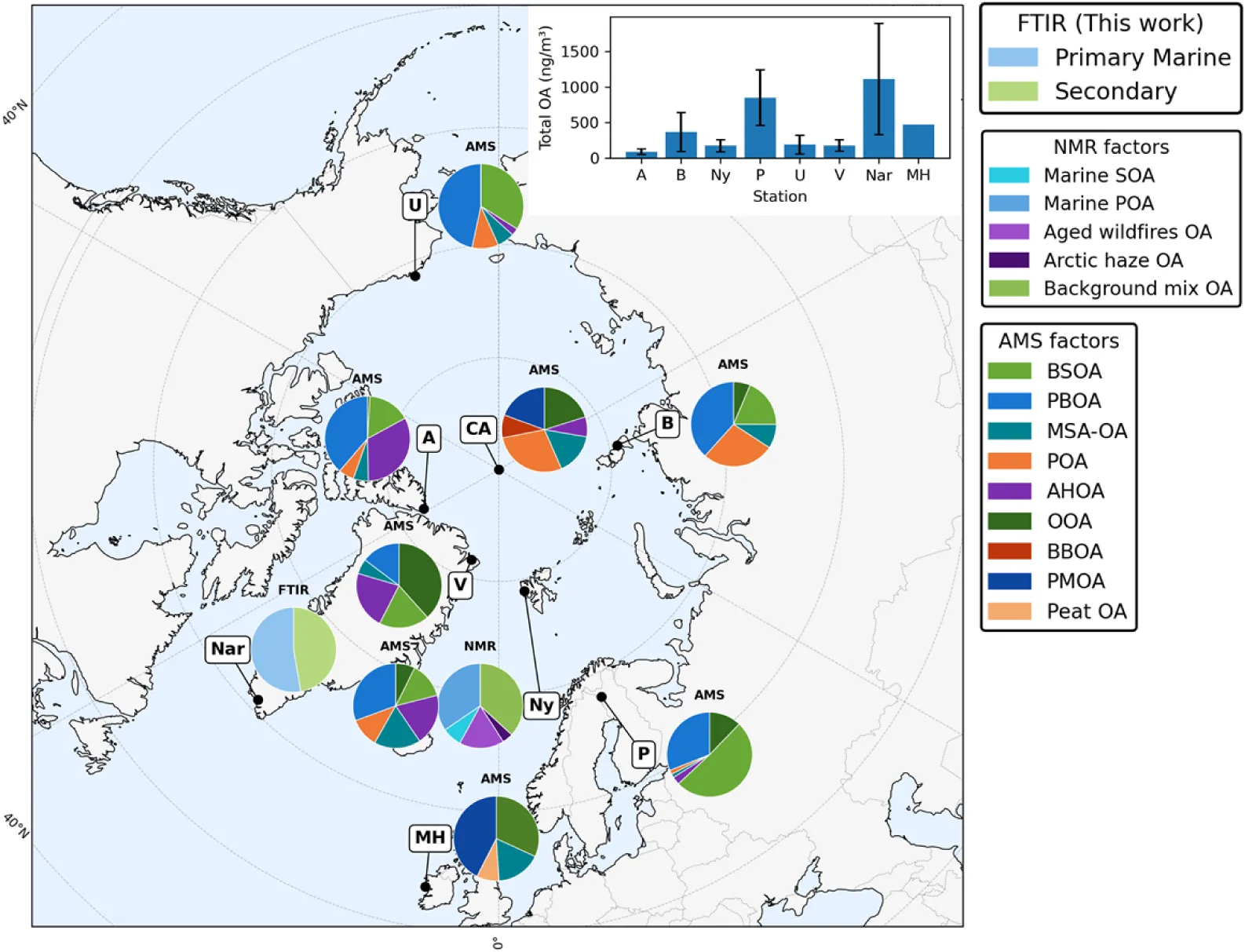
Overview figure of the summertime chemical composition at Narsaq compared to Arctic and coastal sites. The inset shows mean total OA mass concentrations at each station and standard deviations. Narsaq data is from this study while other data is from literature, see Supporting Text S1 for a detailed explanation. A: Alert, B: Cape Baranova, Ny: Ny-Ålesund, P: Pallas, U: Utqiaġvik, V: Villum Research Station, Nar: Narsaq, MH: Mace Head, CA: Central Arctic. BSOA: Biogenic SOA, PBOA: Primary Biological OA, POA: Primary OA, AHOA: Arctic Haze OA, OOA: Oxygenated OA, BBOA: Biomass Burning OA, PMOA: Primary Marine OA.

The chemical composition at Narsaq also features some similarities to that measured in the high Arctic (Figure 6). A factor assigned to MSA-related OA was identified at coastal stations in the high Arctic (Alert, Ny-Ålesund, Utqiaġvik, Cape Baranova, Villum Research Station) using AMS measurements, making up a median 6–18% of total OA,^19^ and 16% of OA over the central Arctic ocean.^18^ The MSA-related factor fractions reported at these sites are smaller than the Secondary factor identified in our study (47%); however, it is possible that not the entirety of our Secondary factor is MSA-related, as discussed in the previous section. Similarly, AMS-based source apportionment at Arctic sites also resolves an oxygenated OA (OOA) factor (median 0–38%, depending on location) that could not be attributed to a specific source due to its highly oxidized character and the absence of distinguishing tracer ions. It is therefore likely that a portion of the AMS OOA signal is also captured within our Secondary factor which contains a high abundance of carboxylic acid and carbonyl functional groups.

Similarly to Narsaq, a primary factor pertaining to sea spray organics was identified over the central Arctic ocean^18^ (19% of June AMS OA mass) and at Ny-Alesund using nuclear magnetic resonance (NMR) spectroscopy, accounting for 35% of the summertime OA mass.^51^ Nevertheless, this sea spray related factor is not present in the analysis of Moschos et al.,^19^ where the major primary OA source is attributed to biological particles. This may be because sea spray aerosol was only important at coastal stations, and therefore not resolved as a factor in the multisite PMF analysis which also comprises land-based stations with relatively small sea spray contributions. In contrast to Narsaq, continental Arctic stations also have significant contributions from regional nonmarine sources, such as biogenic SOA from forests at Pallas, or anthropogenic primary organics from gas flaring at Cape Baranova.^19^ At high Arctic stations (e.g., Villum, Ny-Alesund, Alert), a source of organics related to long-range transported oxidized material (Arctic haze) was observed to contribute even in summer months, although it is typically a winter phenomenon.^52^ This is likely because at high Arctic stations, where total OA concentrations are low, even a minor contribution from long-range transported material represents a detectable fraction of total OA. In contrast, at Narsaq the much larger biogenic marine OA signal means a comparatively minor anthropogenic contribution is not statistically resolved as a separate factor by PMF.

## 4 Implications

Frequent summertime NPF events observed in the area may not only act as a source of new particles, but also affect the size and chemical composition of preexisting particles through condensational growth. During the NPF case study presented in this work, preexisting particles grew up to 80 nm, a size range consistent with reported CCN activation diameters in Arctic marine clouds (58–78 nm).^53^ Particle growth past ∼50 nm has been directly linked to increases in CCN concentration in West Greenland,^54^ consistent with enhancement in CCN concentration observed after growth events locally at Narsaq.^11^ The particle growth was accompanied by a compositional shift toward more hygroscopic species like ammonium sulfate over organics (Figure 5c), acting in the same direction to further support enhanced CCN activity. Given the high frequency of these events throughout multiple summer campaigns,^11^ this pathway is expected to represent a recurring contribution to the regional CCN budget, with direct implications for cloud properties and radiative forcing in this fjord environment.

OA mass concentrations in the studied fjord system are higher than both high Arctic and midlatitude coastal reference sites, with a chemical composition more closely resembling the latter. The high OA loadings suggest that fjord environments can be disproportionately strong sources of aerosol mass, consistent with model studies suggesting that additional marine secondary organic aerosol sources are needed to reconcile simulated and observed Arctic particle size distributions.^22^ Marine-terminating glacier fjords occur throughout the Arctic coastline, suggesting these findings likely extend beyond this specific location. Marine-terminating glacier fjords have not yet been taken into account as a significant source of aerosols in general and CCN specifically, particularly in summer.^5^ The CCN concentrations in summer in the Arctic are very low^55–58^ and it has been shown that cloud formation is highly sensitive to the availability of CCN.^58,59^ At the same time numerical models struggle significantly to represent Arctic cloud formation and the resulting radiative impacts.^60^ Therefore, understanding this potentially important source of CCN locally in the Arctic is critical. Consequently, future measurements at other Arctic fjord systems would help further establish whether the aerosol characteristics identified in Narsaq are representative of fjord environments more broadly, and whether the fjord’s aerosol productivity depends on the state of the cryosphere, i.e., whether marine-terminating glaciers are a critical factor.

Our study identifies that the majority of aerosol is of marine origin (though with a notable contribution of ammonium from e.g., livestock farming, bird colonies, neutralizing sulfate from DMS oxidation) and is therefore directly impacted by fjord dynamics. In particular, with a warming Arctic, marine-terminating glaciers are retreating, negatively impacting ocean biological productivity.^6^ As reduced biological activity would be expected to lower emissions of NPF precursors such as DMS, this decline may in turn reduce the frequency of NPF events and their associated contribution to the regional CCN budget, as well as overall OA loading. Future work should therefore aim to directly measure CCN and precursor gas concentrations (e.g., DMS, ammonia, amines) to more conclusively establish the sources and climate-relevant properties of fjord aerosol identified in this study.

## Supplementary Material



## Notes

Maps of study location; example of an exhaust-polluted spectrum; diagnostics for selecting number of clusters; diagnostics for selecting number of factors; sensitivity test for assigning peaks to amines/carboxylates; cluster assignment counts for 2024 and 2025; meteorological variables associated with each cluster; satellite chlorophyll-a concentrations; fingerprint region comparison of factors; contribution of factors to clusters; case study of chemical composition changes; detailed description of comparison of Narsaq source apportionment results with literature data (PDF)
