# Supplementary material for: Summertime Aerosol Composition in Southern Greenlandic Fjords: Impacts of Sources and New Particle Formation Events
Source: Environ Sci Technol. 2026 Sep 28;60(40):28983–93. doi: 10.1021/acs.est.6c09104 (PMC13674071; doi:10.1021/acs.est.6c09104)
Supplement: Supplementary file 1 [file es6c09104_si_001.pdf]

Supplementary information for:

Summertime aerosol composition in southern Greenlandic fjords: impacts of sources and new particle formation events

Mihnea Surdu[1], Roman Pohorsky[1], Satoshi Takahama[2], Julia Schmale[1]

[1]Extreme Environments Research Laboratory, École Polytechnique Fédérale de Lausanne, Sion, 1951, Switzerland
[2]Laboratory of Environmental Spectrochemistry, École Polytechnique Fédérale de Lausanne, Lausanne, 1015, Switzerland

This file includes:

Figures S1-S10

Supplemetary text S1 and S2.

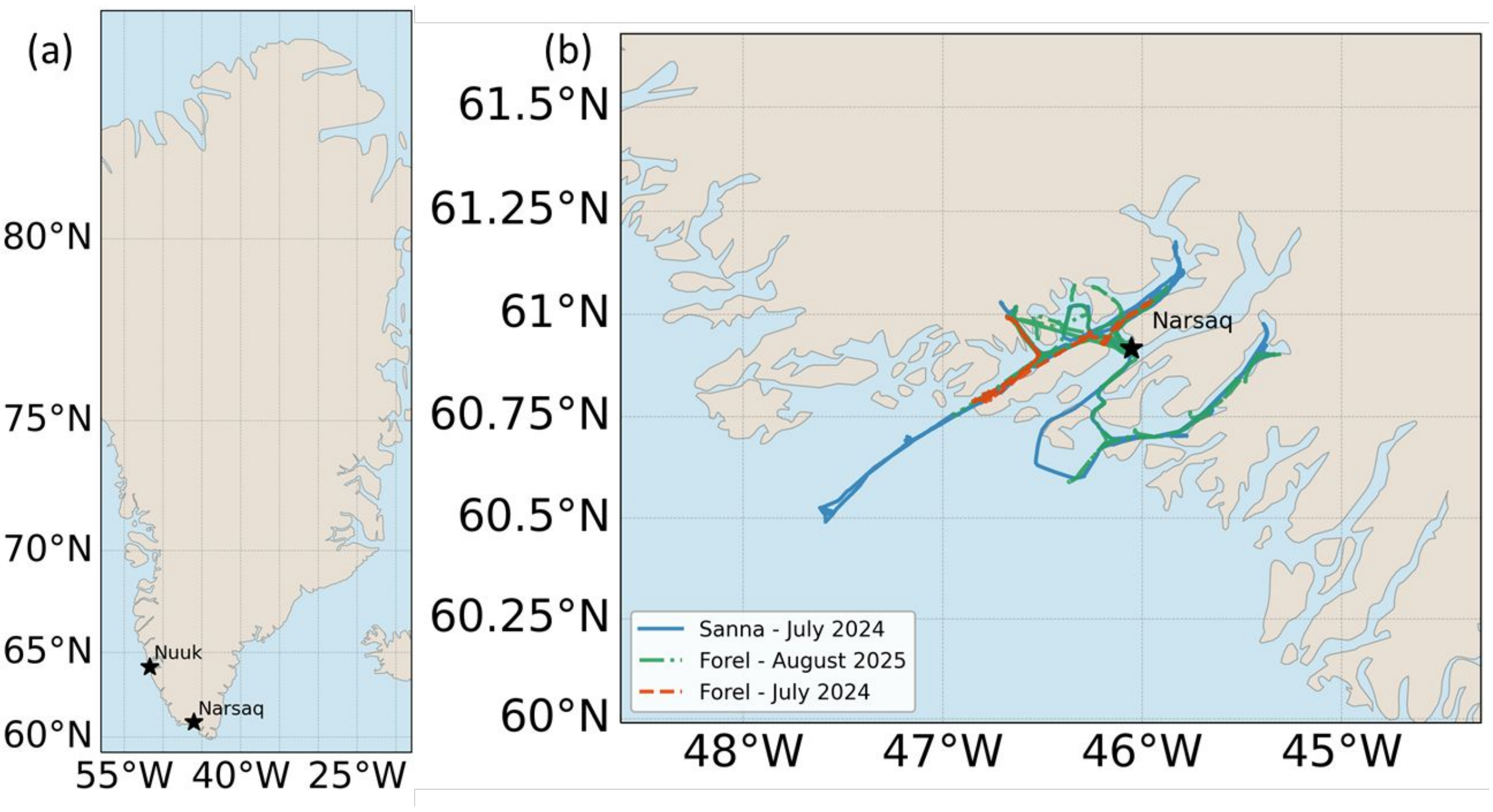

Figure S1. Maps of study location. Panel (a) shows Narsaq's location within Greenland and panel (b) shows the cruise tracks of the three cruise legs.

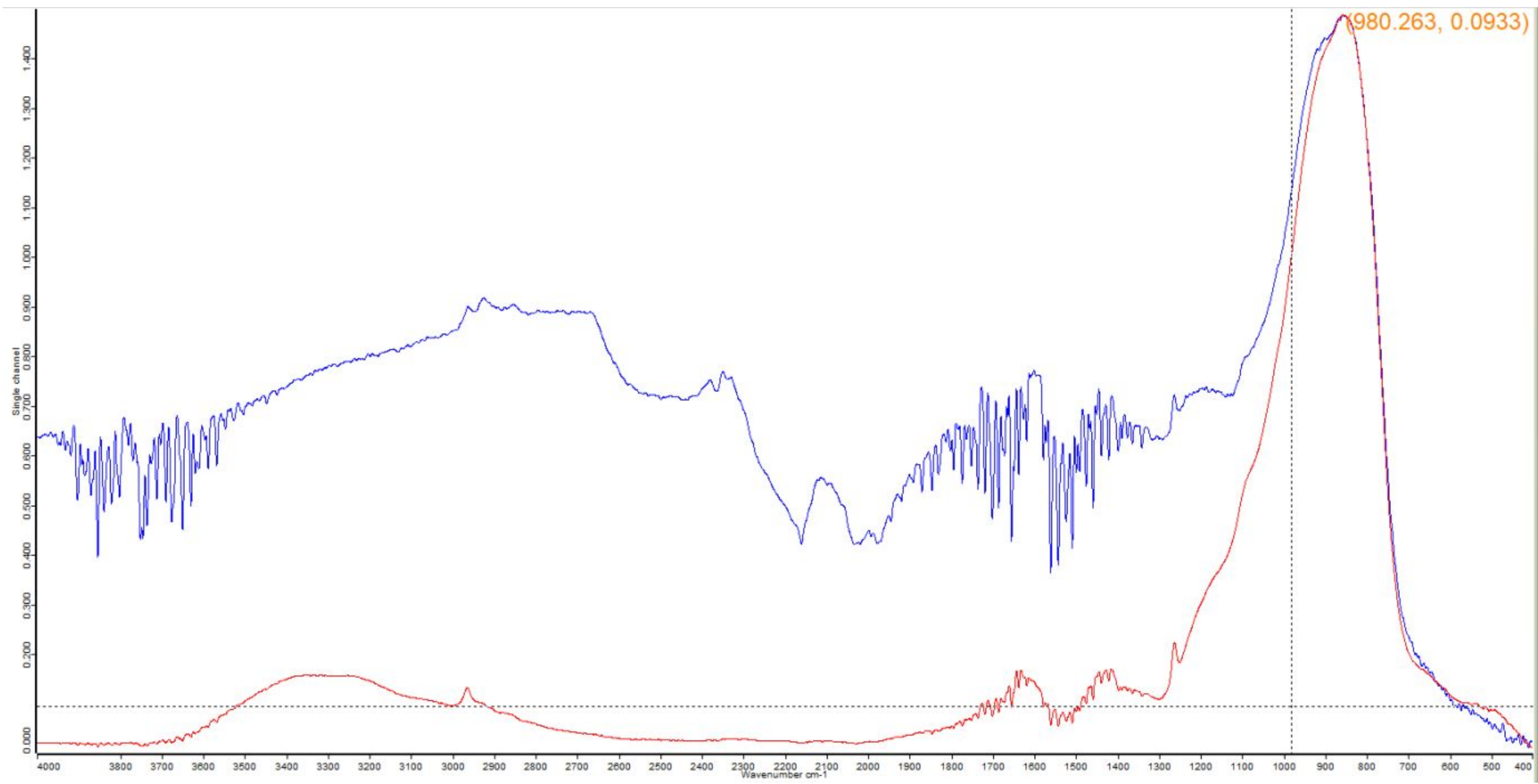

Figure S2. Example of an exhaust polluted (blue) and clean (red) NEMS-FTIR raw spectrum. The polluted spectra are identifiable by broad elevated absorbance increasing with higher wavenumber (characteristic of black carbon absorption in the near infrared). This feature is not present in the clean spectra.

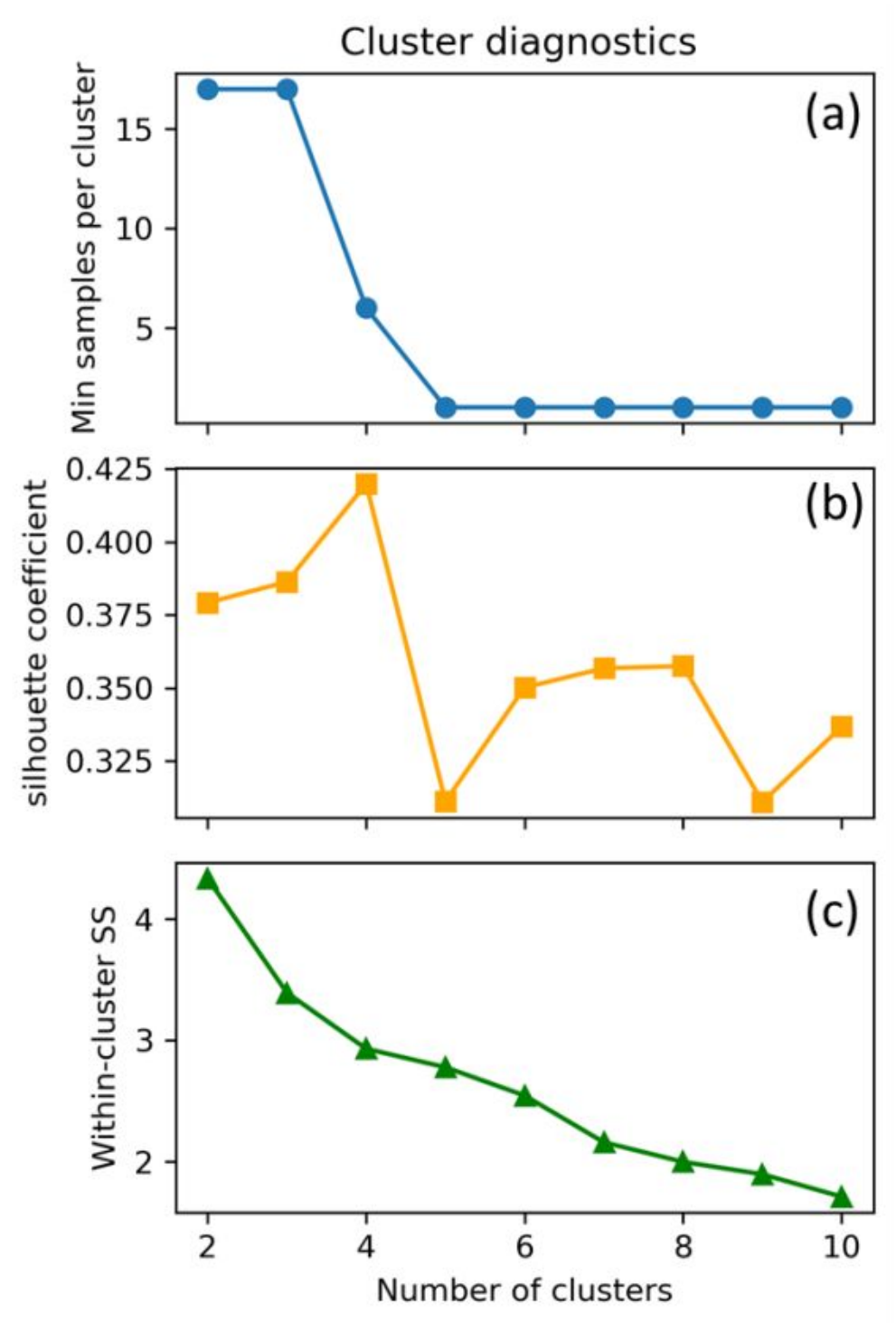

Figure S3. Diagnostics for selecting the optimal number of clusters. Minimum samples per cluster, silhouette coefficient and within-cluster sum of squares in panels (a), (b) and (c), respectively.

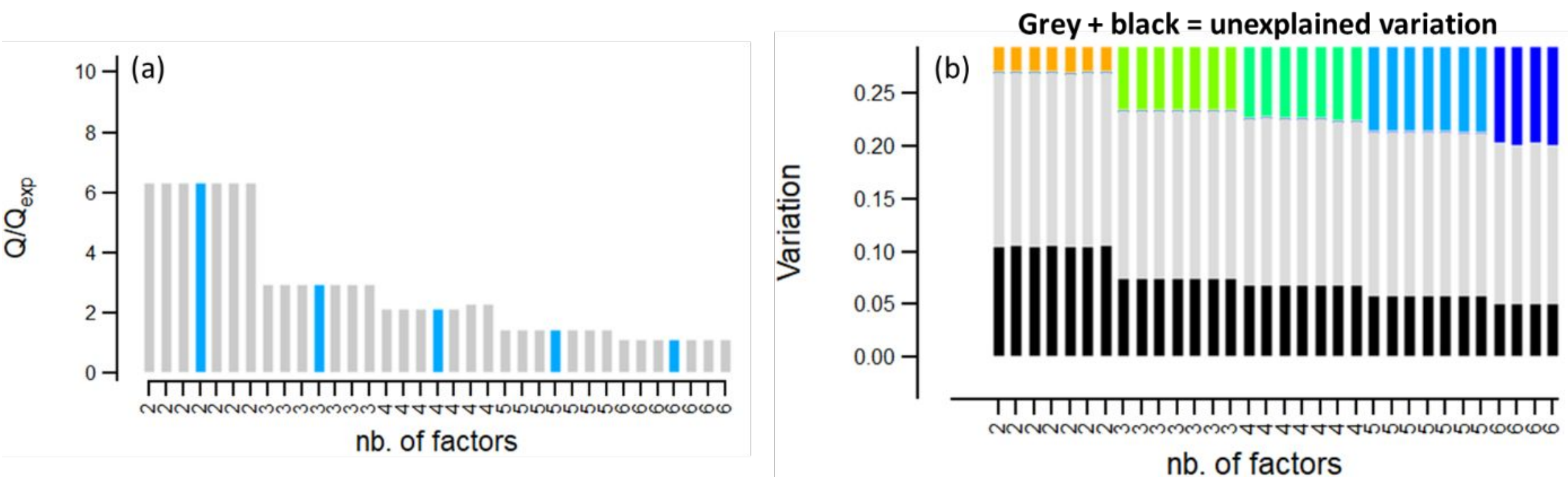

Figure S4. Diagnostics for selecting optimal number of factors by positive matrix factorization. Panel (a) shows Q/Q_{exp} and panel (b) shows the unexplained variance of the solution. The repeat solutions with the same number of factors represent changes in the rotational parameter FPEAK.

Supplementary Text S1 – sensitivity test for assigning 1630 cm^{-1} peak to amines/carboxylates

To evaluate whether the absorbance at 1630 cm^{-1} attributed to amines could instead arise from overlapping carboxylate absorption, we performed a sensitivity analysis in which the amine contribution to each spectrum was fixed to the amine-to-alcohol ratio reported for primary sea spray aerosol by Frossard et al. (2014)[1]. The residual absorbance at 1630 cm^{-1}, after subtracting this fixed amine contribution, was assigned entirely to carboxylates using the molar absorption coefficient reported by Boris et al. (2019)[2] for ammonium oxalate. The resulting functional group composition of each cluster under this scenario is shown in Figure S5.

Under this scenario, carboxylate concentrations exceed the separately quantified carboxylic acid concentrations by a factor of 2.8 (campaign average). The combined carboxylic acid + carboxylate contribution reaches 25% of organic matter on campaign average. This value is considerably higher

than small organic acid contributions reported at other marine and polar sites, scaled to the median organic matter concentration: mean 0.4% for the Southern Ocean, 2.7–6.3% for the Arctic Ocean, and roughly 5% on average for the western Pacific.[3,4]

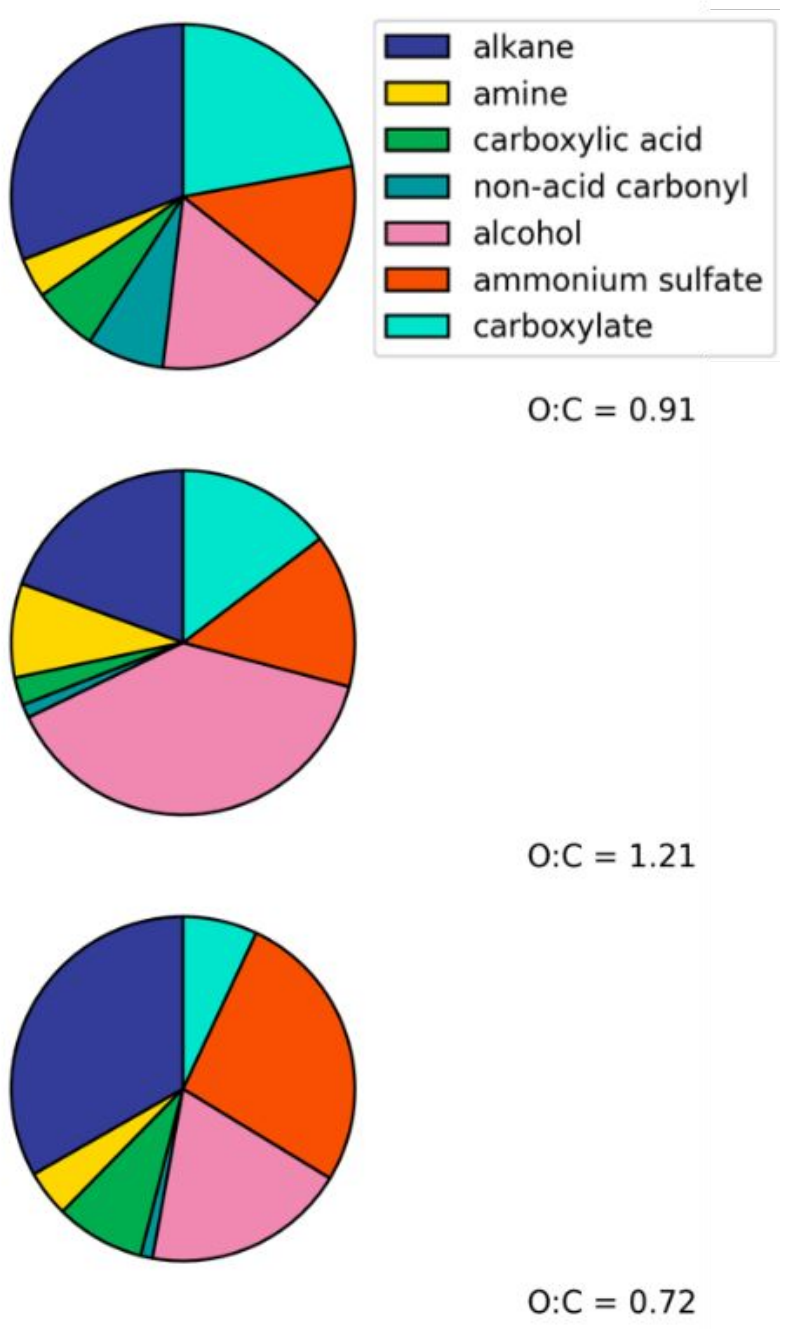

Figure S5. Organic functional group and ammonium sulfate contributions to each cluster under the sensitivity test described in Text S1 – Cluster 1 (top), Cluster 2 (middle), Cluster 3 (bottom).

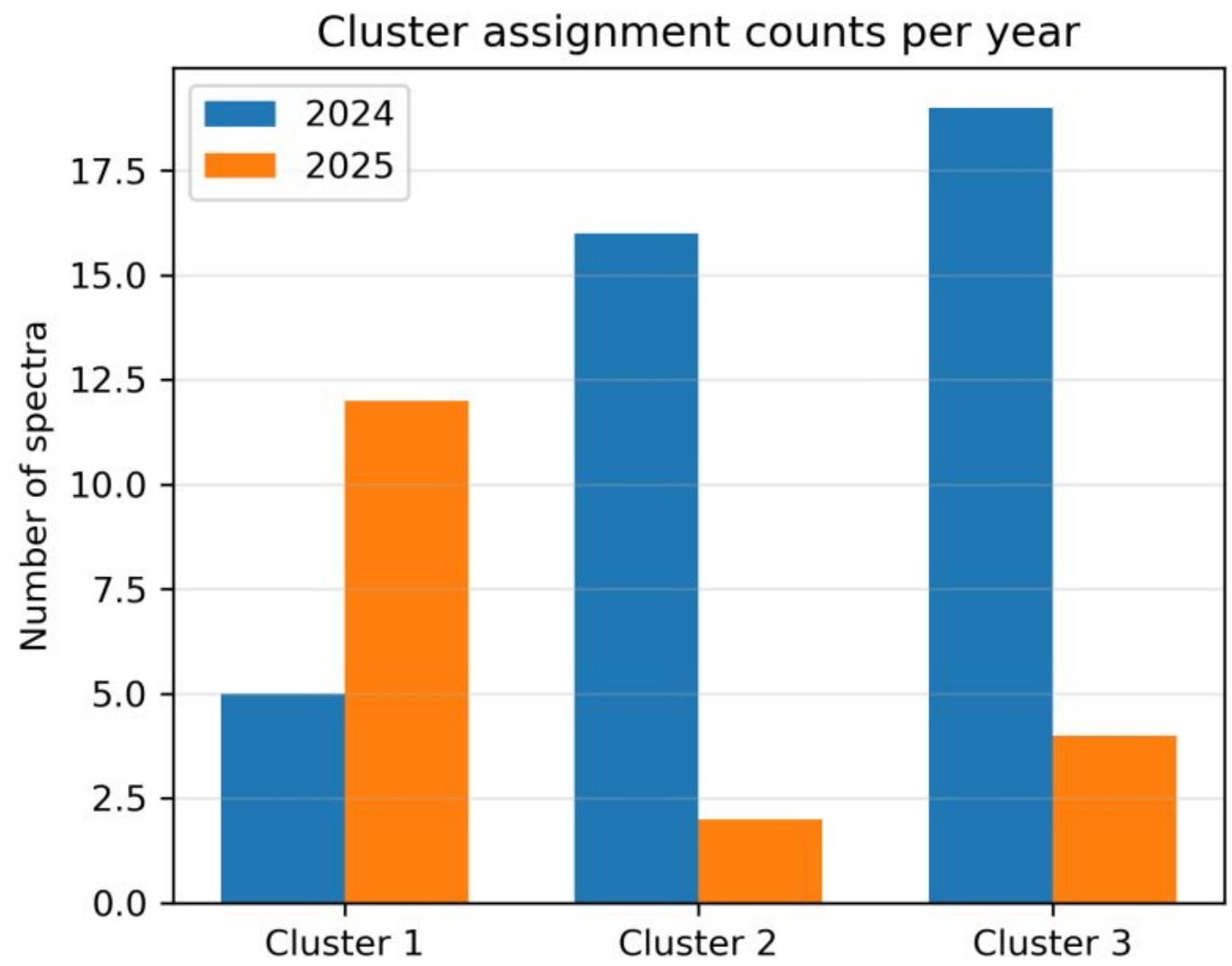

Figure S6. Cluster assignment counts for the July 2024 and August 2025 campaigns.

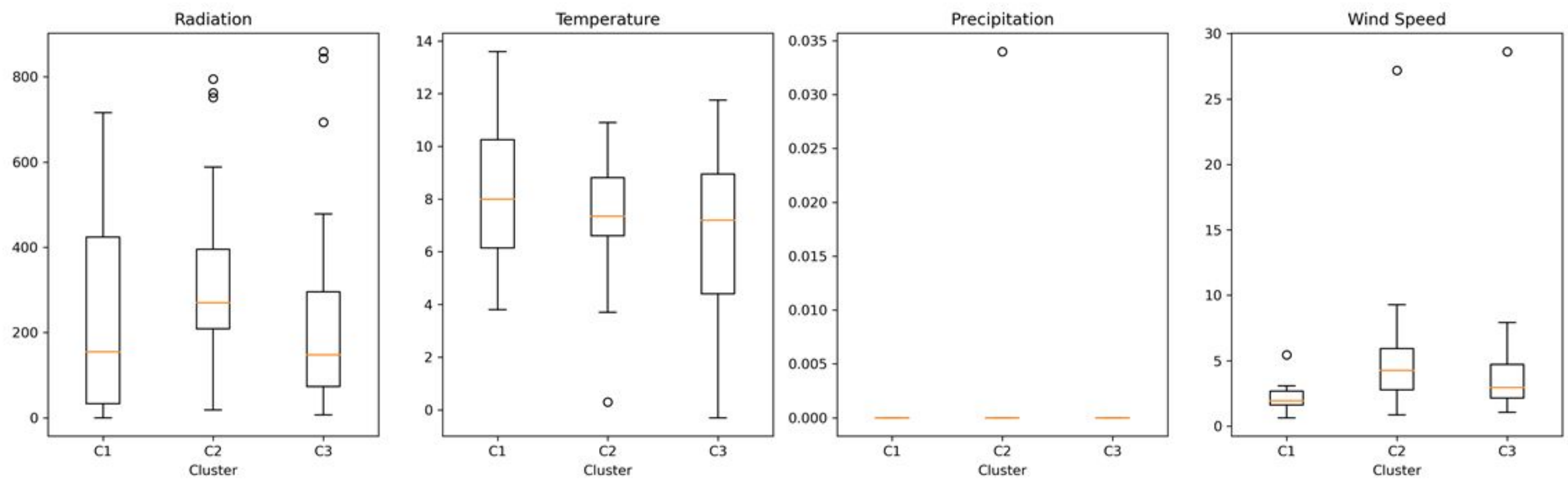

Figure S7. Meteorological variables associated with each assigned cluster. Radiation in W/m^2, Temperature in degrees Celsius, Precipitation in mm and Wind Speed in m/s.

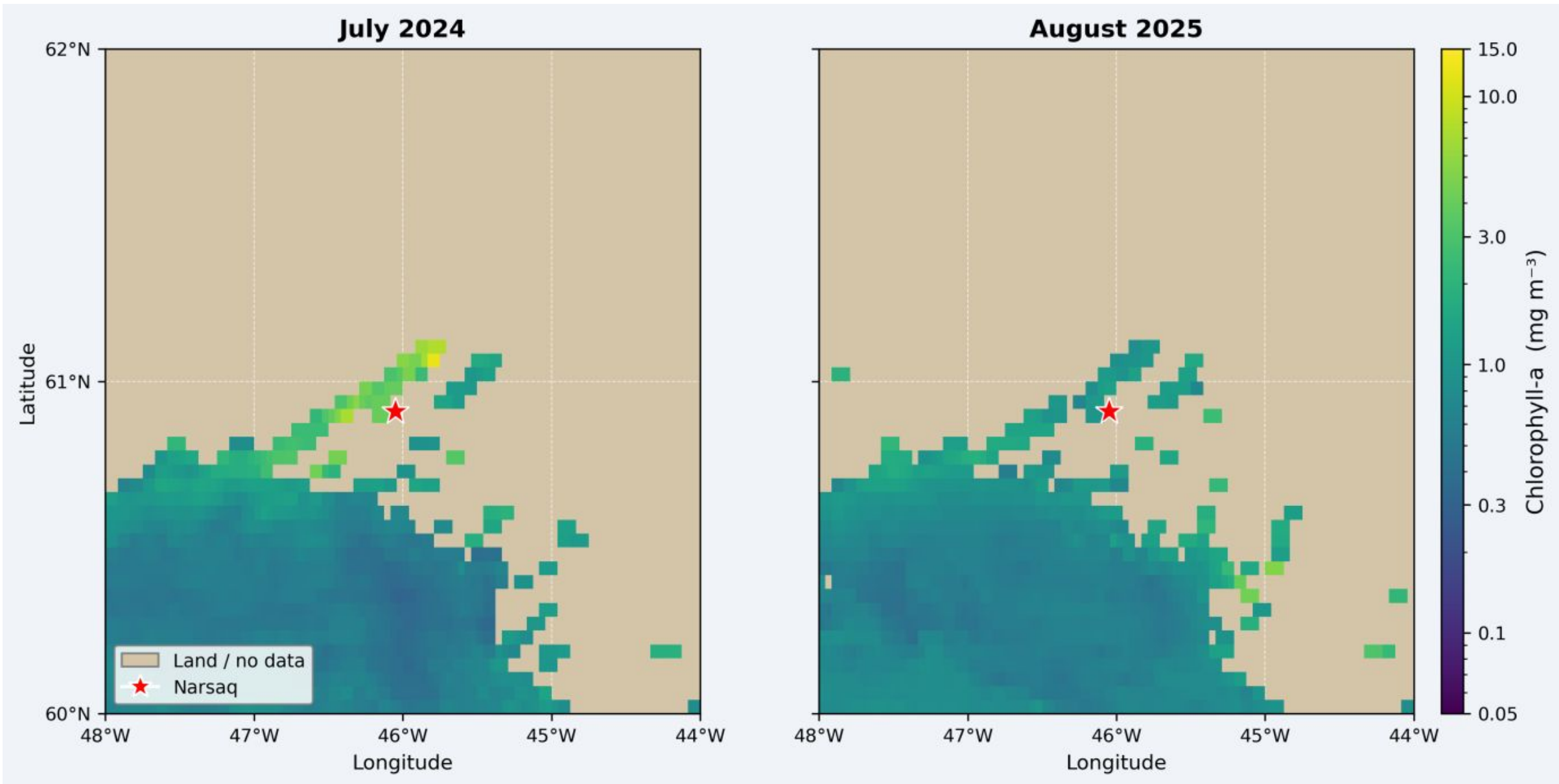

Figure S8. Monthly average chlorophyll-a concentrations over the Narsaq region, obtained from MODIS-Aqua (https://oceancolor.gsfc.nasa.gov/l3). Layer 3, mapped data was used at 4km resolution. Grid cells over land, or with no datapoints (e.g. due to cloud cover) are coloured beige.

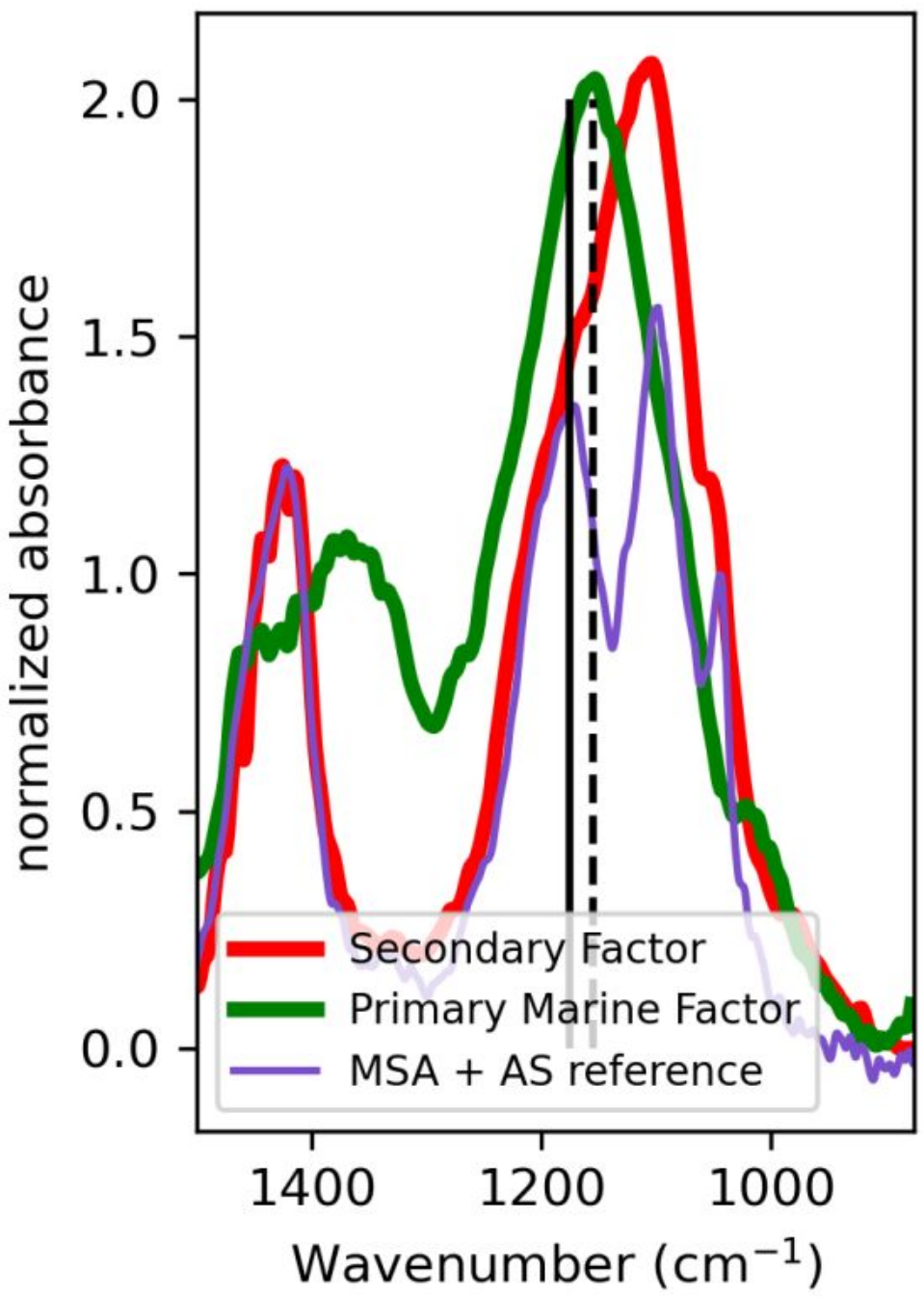

Figure S9. Fingerprint region comparison of the two PMF factor profiles with the linear combination of spectra of standard pure methane sulfonic acid and ammonium sulfate (MSA+AS). The solid and dashed vertical lines represent peak positions at 1175 and 1155 cm^{-1}, assigned to MSA and C-O vibrations, respectively.

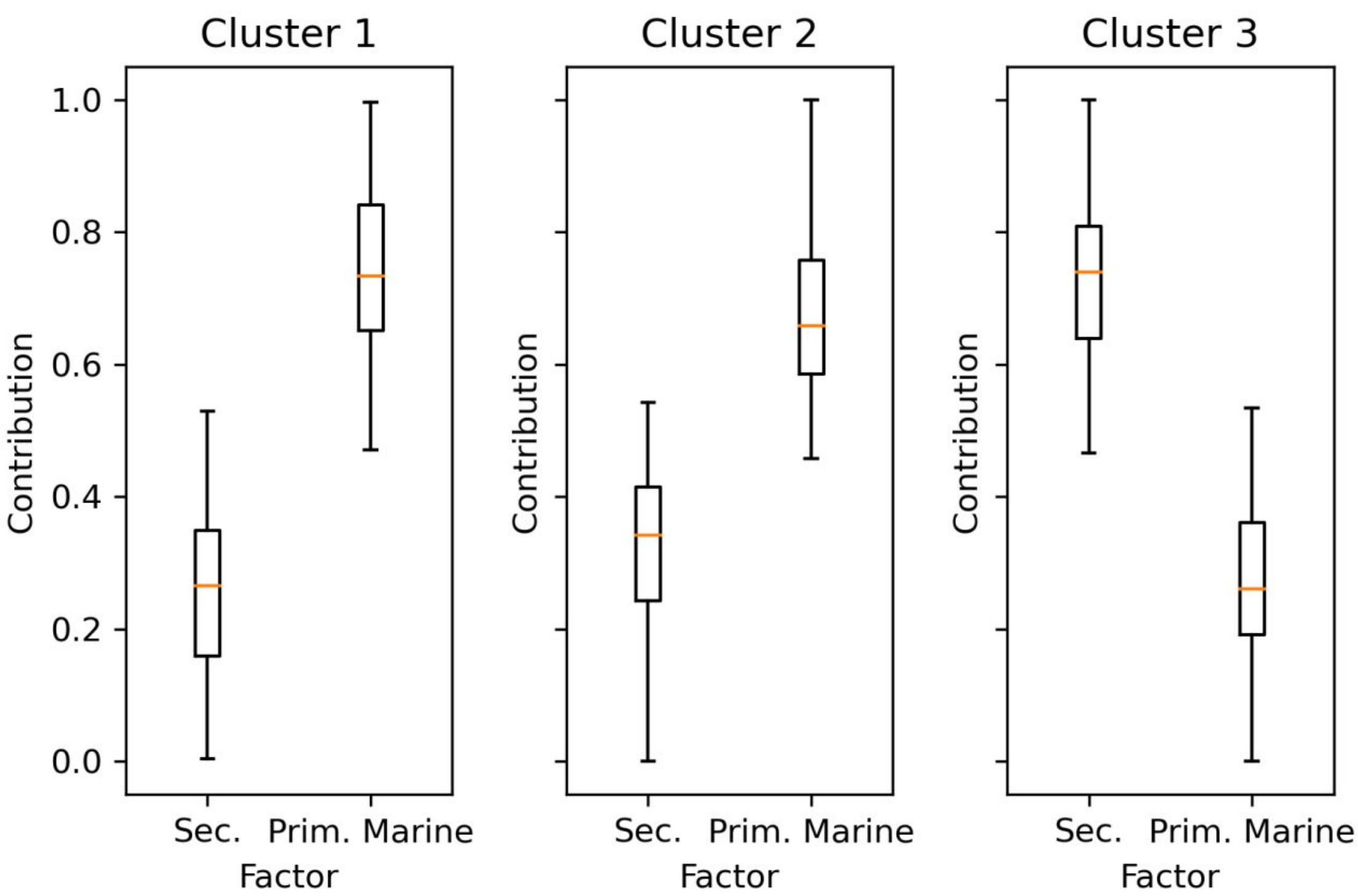

Figure S10. Contribution of PMF factors (Sec. for Secondary, Prim. Marine for Primary Marine) to each cluster.

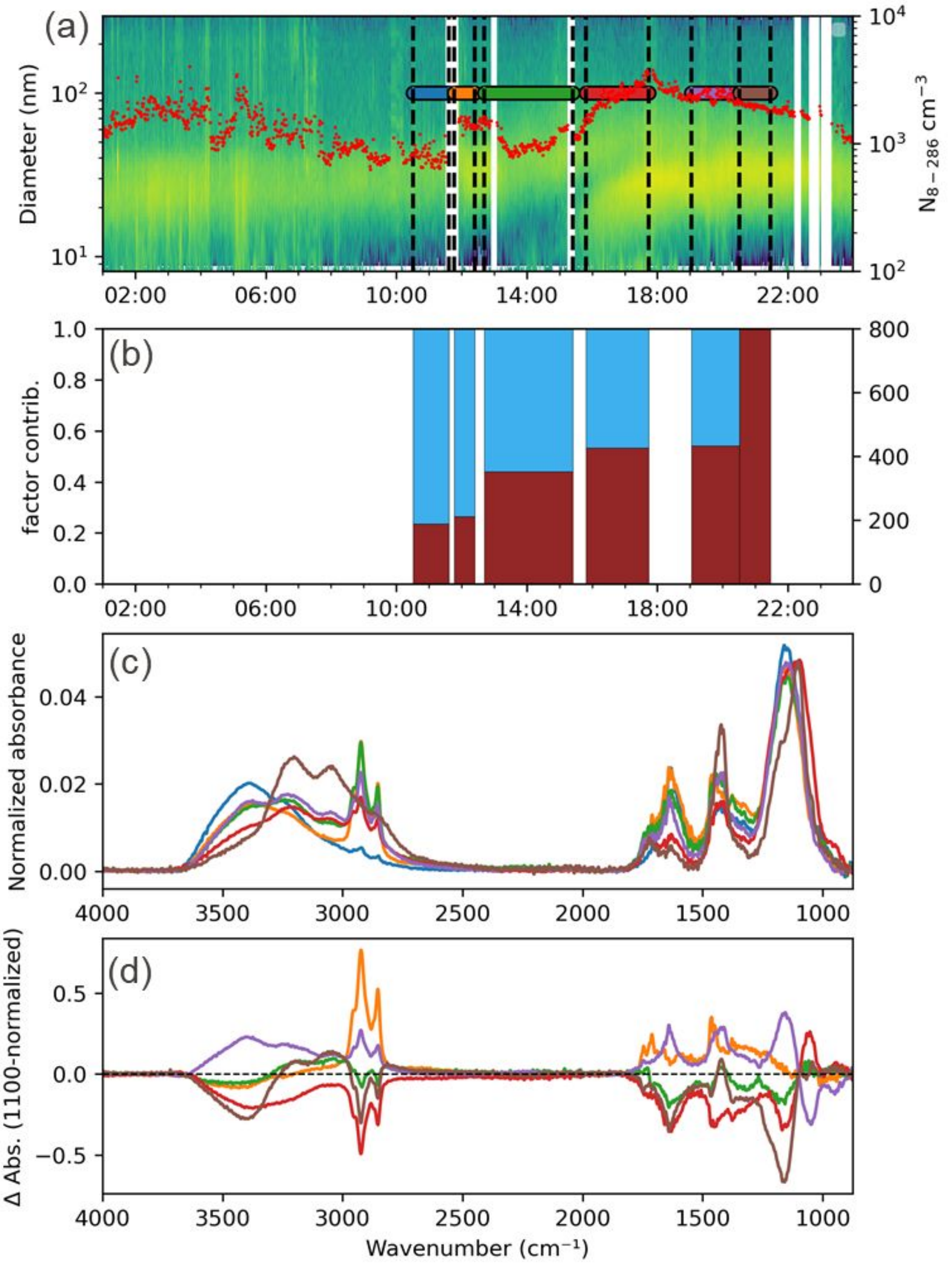

Figure S111. Case study of chemical composition change during an NPF event. Same as Figure 5, but with the addition of difference spectra in panel **(d)**. Difference spectra are normalized to the sulfate peak at 1100 cm^{-1}, and coloured by the colour of the endpoint spectrum in **(c)**. For example, the difference between the blue and orange spectra in **(c)** is coloured orange in **(d)**.

Supporting Text S2. Detailed description of comparison of Narsaq source apportionment results to literature data.

AMS data for stations Alert, Cape Baranova, Pallas, Utqiaġvik and Villum Research Station was used from the offline-AMS PMF study of Moschos et al. (2022)[5]. AMS Mace Head data obtained from online-AMS PMF study of Chevassus et al. (2025)[6]. AMS Data over the central Arctic Ocean is from the study of Heutte et al. (2025)[7]. AMS Data at Ny-Alesund station represents the mean of Gruvebadet and Zeppelin observations from Moschos et al., and data from Paglione et al. (2025)[8]. In order to enable a fair comparison to our observational data in Narsaq, only observations during the months of July and

August were considered across all datasets, with the exception of the AMS data over the central Arctic Ocean where summer data was only available for June. The median of all samples in the above time period was taken and is plotted in Figure 6. In our study, a total suspended particulates (TSP) inlet was used for particle collection. TSP samples were taken at Alert, Gruvebadet and Utqiaġvik while PM_{10} samples were collected at Cape Baranova, Pallas, Villum Research Station and Zeppelin in the study of Moschos et al. (2022). An AMS with submicron measurement capabilities was used by Chevassus et al. (2025) and Heutte et al. (2025) for online measurements, and PM_1 samples were collected during the study of Paglione et al. (2025).
